# Supplementary material for: 3D Spheroid Human Dermal Papilla Cell as an Effective Model for the Screening of Hair Growth Promoting Compounds: Examples of Minoxidil and 3,4,5-Tri-O-caffeoylquinic acid (TCQA)
Source: Cells. 2022 Jun 30;11(13):2093. doi: 10.3390/cells11132093 (PMC9265566; doi:10.3390/cells11132093)
Supplement: Supplementary file 1 [file cells-11-02093-s001.zip › Supp Figures 2022.pptx]

## Slide 1
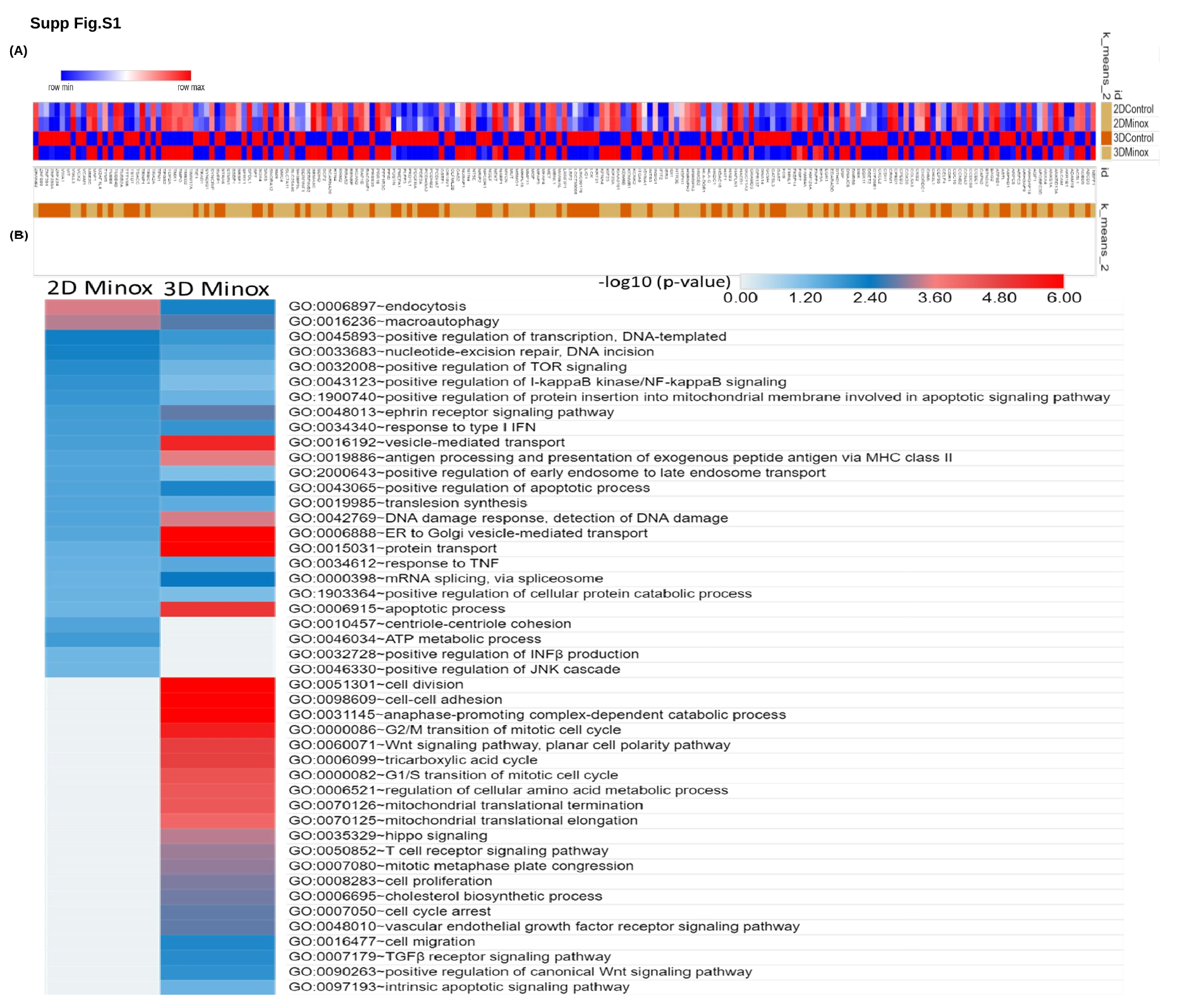

Supp Fig.S1
(A)

## Slide 2
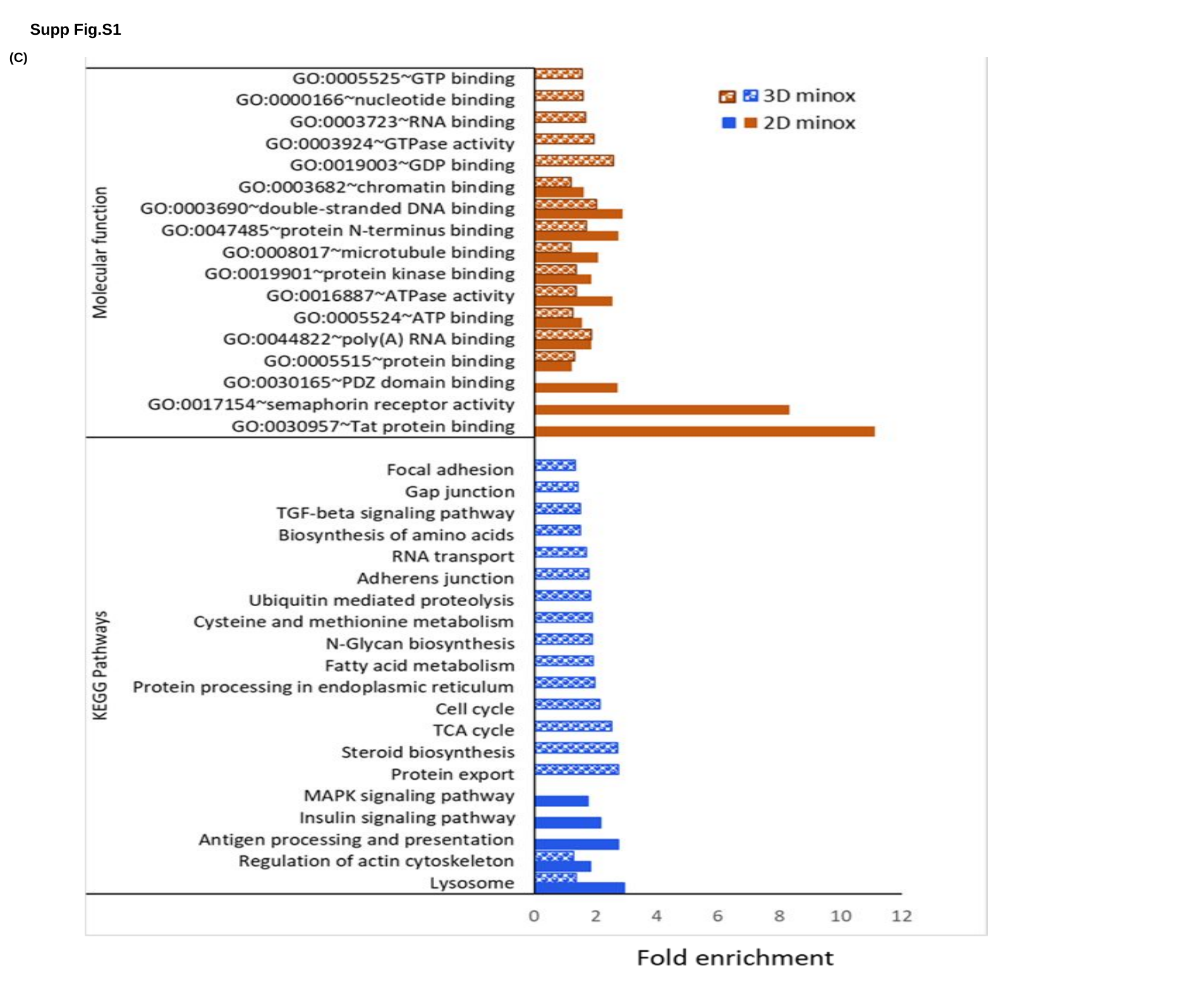

Supp Fig.S1
(C)

## Slide 3
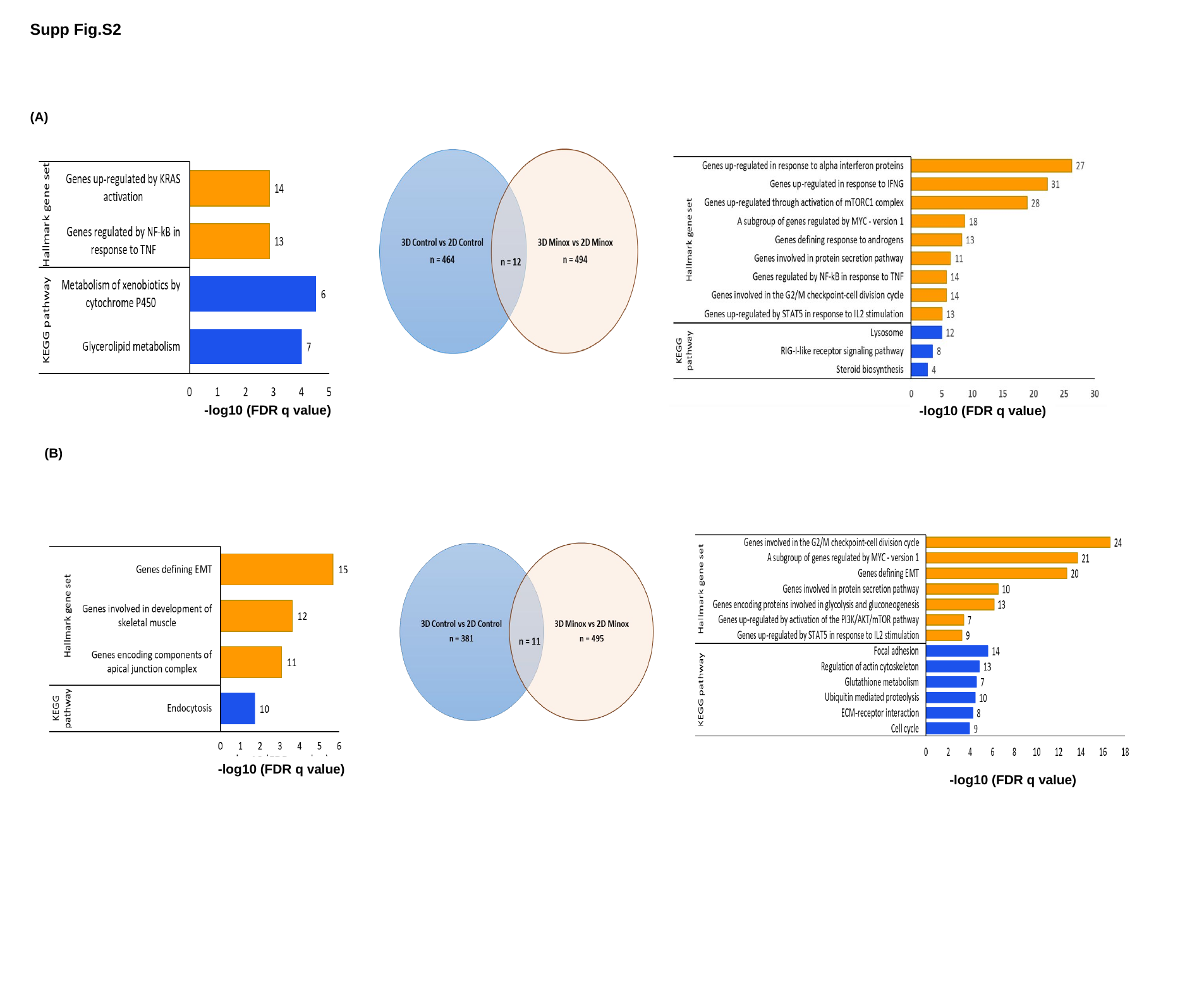

Supp Fig.S2
(A)
-log10 (FDR q value)
-log10 (FDR q value)
(B)
-log10 (FDR q value)
-log10 (FDR q value)
