# Supplementary material for: 3D Spheroid Human Dermal Papilla Cell as an Effective Model for the Screening of Hair Growth Promoting Compounds: Examples of Minoxidil and 3,4,5-Tri-O-caffeoylquinic acid (TCQA)
Source: Cells. 2022 Jun 30;11(13):2093. doi: 10.3390/cells11132093 (PMC9265566; doi:10.3390/cells11132093)
Supplement: Supplementary file 1 [file cells-11-02093-s001.zip › Supp Tables.pdf]

Supp Table S1: Top upregulated genes in 2D minoxidil-treated cells (vs 2D control) *.

Gene symbol	Gene name	Biological function	Fold-change	*P* value **
CTNNB1	Catenin (cadherin associated protein), beta 1	Anagen phase regulation; hair follicle morphogenesis; positive regulation of fibroblast growth, dermal papilla cells proliferation	11.06	0.049
COL5A	Collagen, type V, alpha	Fibrillogenesis in the skin and the hair follicle	8.54	0.011
FGF7	Fibroblast growth factor 7	Hair follicle morphogenesis; hair growth cycle regulation	6.16	0.022
STAT1	Signal transducer and activator of transcription 1	Anagen onset; hair growth regulation	4.83	0.023
BMP1	Bone morphogenetic protein	Skin development; hair follicle growth	4.59	0.018
BMPER	BMP binding endothelial regulator	Hair follicle development and morphogenesis	3.11	0.001
FSTL1	Follistatin-like 1	Hair wave propagation	3.03	0.012
WLS	Wntless	Wnt pathway	2.92	0.016
FGF5	Fibroblast growth factor 5	Hair follicle morphogenesis ; hair growth cycle regulation	2.06	0.010
ALPL	Alkaline phosphatase	Dermal papilla marker	2.03	0.058

Supp Table S2: Top downregulated genes in 2D minoxidil-treated cells (vs 2D control) *.

**Gene symbol**	**Gene name**	**Biological function**	**Fold-change**	***P* value ****
MMP24	Matrix metallopeptidase 24	Collagen breakdown; hair follicle and skin aging	-7.52	0.040
Defb8	Defensin, beta 8	Upregulation during telogen phase; absent during anagen phase	-6.6	0.032
MMP7	Matrix metallopeptidase 7	Collagen breakdown; hair follicle and skin aging	-6.47	0.001
STK24	Serine/threonine-protein kinase 24	Promotion of tumor progression	-5.72	0.032
HYAL1	Hyaluronidase-1	Tumor progression	-5.24	0.017
METTL21B	EEF1A lysine methyltransferase 3	Oxidative stress induction	-4.74	0.007
NELL2	Protein kinase C-binding protein	MAPK pathway modulation	-4.28	0.012
TAX1BP3	Tax1-binding protein 3	Wnt/β-Catenin repression	-4.22	0.042
WNK2	Serine/threonine-protein kinase	Negative regulation of MAPK pathway	-4.13	0.008
MMP8	Matrix metallopeptidase 8	Collagen and filament degradation	-4.08	0.045

Supp Table S3: Top DEGs in 2D culture (TCQA vs Control) *.

Gene symbol	Gene name	Biological function	Fold-change
WNT11	Wingless-type MMTV, member 11	Wnt pathway	8.25
VCAN	Versican	Cell aggregation marker, Cell adhesion and proliferation	4.61
FGF5	Fibroblast growth factor 5	Hair follicle morphogenesis; hair growth cycle regulation	3.98
COL3A1	Collagen, type III, alpha 1	Fibrillogenesis in the skin and the hair follicle	3.40
SOX4	SRY (sex determining region Y)-box 4	Hair regeneration	3.26
BMP4	Bone morphogenetic protein 4	Control hair follicle growth and development	2.47
Aebp1	AE binding protein 1	Regulator of telogen hair follicle	-2.46
Satb1	Special AT-rich sequence binding protein 1	Regulator of transcription during telogen	-2.02
Egr1	Early growth response 1	Negative regulation of Wnt/β-Catenin; upregulated with aging	-1.7
AXIN2	Axis inhibition protein 2	Wnt-responsive gene	-1.5
Gsk3b	Glycogen synthase kinase 3 beta	Phosphorylation of β-catenin	-1.5

Supp Table S4: Top upregulated hair growth-related genes 3D culture (TCQA vs Control) *.

**Gene symbol**	**Gene name**	**Biological function**	**Fold-change**
BMPR-1A	Bone morphogenetic protein	Skin development; hair follicle growth	64.89
CTNNB1	Catenin (cadherin associated protein), beta 1	Anagen phase regulation; hair follicle morphogenesis; positive regulation of fibroblast growth, dermal papilla cells proliferation	43.57
WNT11	Wingless-type MMTV, member 11	Wnt pathway	40.98
COL3A1	Collagen, type III, alpha 1	Fibrillogenesis in the skin and the hair follicle	28.23
FGF1	Fibroblast growth factor 1	Hair follicle morphogenesis; hair growth cycle regulation	26.45
FGF5	Fibroblast growth factor 5	Hair follicle morphogenesis; hair growth cycle regulation	23.61
WLS	Wntless	Wnt secretion and pathway	16.38
NRAS	Neuroblastoma RAS viral oncogene homolog	Cell proliferation	16
SOX4	SRY (sex determining region Y)-box 4	Hair regeneration	13.92
VCAN	Versican	Cell aggregation marker,<br>Cell adhesion and proliferation	12.21
ALPL	Alkaline phosphatase	Wnt/β-catenin pathway regulator	8
BMP4	Bone morphogenetic protein 4	Control hair follicle growth and development	2.47

Supp Table S5: Top downregulated hair growth-related genes 3D culture (TCQA vs Control) *.

Gene symbol	Gene name	Biological function	Fold-change
Gsk3b	Glycogen synthase kinase 3 beta	Phosphorylation of β-catenin	-6
Tcf3	Transcription factor 3	Wnt/β-Catenin repression	-5
AXIN2	Axis inhibition protein 2	Wnt-responsive gene	-5
Nfatc1	Nuclear factor of activated T cells, cytoplasmic, calcineurin dependent 1	Stem cells quiescence	-4.9
Egr1	Early growth response 1	Negative regulation of Wnt/β-Catenin; upregulated with aging	-4
Dst	Dystonin	Cell cycle arrest	-3
H2afv	H2A histone family	Stem cell quiescence, Anagen delay	-3
Shisa3	Shisa family member 3	Negative regulator of Wnt/β-Catenin	-2.9
LDB3	LIM Domain Binding 3	Wnt-responsive gene; Regulation of hair follicle during telogen	-2.7

For all Tables

*Genes functions were obtained from Mouse Genome Informatics (MGI) and Gene Set Enrichment Analysis (GSEA)

**ANOVA was performed to assess the level of significance between groups. The gene expression was considered significant when fold change was ≥2-fold (control vs TCQA
